# Robert Hooke's Contributions to Hydrogeology

**DOI:** 10.1111/gwat.12849

**Published:** 2018-12-19

**Authors:** David Deming

**Affiliations:** ^1^ College of Arts & Sciences University of Oklahoma 660 Parrington Oval, Norman OK, 73019

## The Unsociable Virtuoso

He was, according to Isaac Newton, “a man of a strange unsociable temper” (Newton 1960, 437). Robert Hooke was known for a “peevish temper” and established a history of conflict with several people (Clerke 1891, 285). He was also a brilliant experimental physicist who made contributions to many fields, including geology and hydrogeology.

Robert Hooke was born on July 18, 1635, on the Isle of Wight. Hooke's biographer, Robert Waller (c. 1660–1715), described the youth in terms partly contradictory. As a child, Hooke was “very infirm and weakly,” yet also “very sprightly and active in running [and] leaping” (Waller 1705a, ii). The hopes of any scholarly pursuits for the sickly child were dashed by the fact that studies of any type gave him headaches. However, the young Hooke soon evidenced a mechanical genius. “There was nothing he saw done by any mechanic, but he endeavored to imitate” (Waller 1705a, ii). Hooke constructed a “working replica” of a clock out of wood and various “ingenious mechanical toys” (Westfall 2008, 481).

Hooke also had an artistic talent for drawing, a skill that later enabled him to produce breathtaking illustrations for the book *Micrographia* (Hooke 1665). The story is told that at some point in Hooke's youth, the painter John Hoskins (c. 1590–1664) visited the Isle of Wight. Upon watching Hoskins work, Hooke asked himself the question, “why cannot I do so too?” (Aubrey 1898, 409). So the child immediately went to work “and made a picture” (Aubrey 1898, 409).

When Hooke was 13 years old, his father died. The youth took his inheritance of £100 and journeyed to London. Hooke intended to apprentice with the Dutch painter, Peter Lely (1618–1680), whose studio was in London. It was said that Lely “liked him [Hooke] very well,” but Hooke soon concluded that Lely had nothing to teach him (Aubrey 1898, 410). Hooke ended up at Westminster School, an institution whose traditions and history date to as early as AD 960. Hooke must have impressed the headmaster of the school, Richard Busby (1606–1695), because Busby boarded Hooke in his own home (Waller 1705a, iii). Busby was infamous for the liberal employment of corporeal punishment at Westminster. “The deliberate breaking of the young child's will … by the harshest physical beating … was thought to be the key to successful child‐rearing in the sixteenth and seventeenth centuries” (Stone 1977, 101). And Busby was “the severest of severe pedagogues … he ruled the school with a rod of birch” (Overton 1886, 30). Yet “it is certain that he [Busby] gained the veneration and affection of many of his pupils” (Barker 1895, 27).

At Westminster, Hooke studied Latin and Greek, and acquired some knowledge of Hebrew and “other oriental languages” (Waller 1705a, iii). It is claimed that Hooke had such a proficiency for mathematics that he mastered the six books of Euclid's geometry in 1 week (Aubrey 1898, 410). But if Hooke had any skill in mathematics, he never displayed or applied it in any of his physics or scientific work. His orientation was entirely experimental, and Hooke never systematically expounded the mathematical consequences of his intuitive insights.

In 1653, Hooke enrolled at Christ's College at Oxford. Here he met the men whose association would propel him into his scientific career and employment by the Royal Society. As early as 1647, Robert Boyle referred to the activities of an “invisible college” in his letters (Birch 1744, 20). This was an informal association of men interested in applying the epistemological methods advocated by Francis Bacon (1561–1626) in *Novum Organum* (Bacon 1620). They met informally at first, but eventually would coalesce as the Royal Society in 1662 (Deming 2012, 209).

At the age of 16 Hooke began to develop a curvature of the spine that appears to have been caused by a disease known as Scheuermann's kyphosis, an affliction in which the vertebrae of the back grow unevenly. The spine becomes curved, mobility is restricted, and the victim suffers from severe and disabling pain (Inwood 2003, 10). If Hooke indeed suffered from Scheuermann's disease, it may explain his reputation for possessing a foul and combative temper. Hooke's spinal curvature grew worse with age. Richard Waller described him as “very crooked” in stature with a “melancholy, mistrustful, and jealous” temper (Waller 1705a, xxvi). Yet Hooke was not without his friends. John Aubrey (1626–1697) admired Hooke as “the greatest mechanic this day in the world” (Aubrey 1898, 411).

Hooke lived before the age of technical specialization in the sciences. Yet even so, he exhibited a wide range of interests. In physics, Hooke is known for *Hooke's law*, the observation that in elastic materials strain is proportional to stress. In Hooke's words, “the rule or law of nature in every springing body is, that the force or power thereof to restore itself to its natural position is always proportionate to the distance or space it is removed therefrom” (Hooke 1678, 4).

Robert Hooke also made contributions to chemistry, astronomy, geology, meteorology, and horology. In 1668, Hooke invented a marine barometer that could withstand the rigor of a sea voyage (McConnell 2005, 88). Among those impressed by Hooke's barometer was Edmond Halley (1656–1742). Halley provided a personal testimonial as to the utility of Hooke's marine barometer. “I had one of these barometers with me in my late southern voyage, and it never failed to prognosticate and give early notice of all the bad weather we had” (Halley 1700, 794).

Along with Christiann Huygens (1629–1695), Hooke established a credible claim to be the inventor of the *balance spring*, a spring attached to the balance wheel of a portable timekeeper that causes the wheel to oscillate at a resonant frequency (Hooke 1676, 26). The balance spring made it theoretically possible to devise a portable timekeeper accurate enough to solve the problem of determining longitude at sea. This dream was realized with the fabrication of John Harrison's (1693–1776) No. 4 chronometer. In a voyage to Jamaica in 1761–62, Harrison's No. 4 only lost 5 s of time in 65 days at sea, an accuracy sufficient to calculate longitude during a sea voyage (Gould 1935).

Hooke was not reluctant to claim credit for everything he did, and much of what he never accomplished. “There was scarcely a discovery made in his time which he did not conceive himself entitled to claim” (Clerke 1891, 286). Hooke's tendency to exaggerate his contributions extended even to Isaac Newton's theory of universal gravitation. In a lecture delivered to the Royal Society in February of 1690, Hooke complained bitterly of “those properties of gravity which I myself first discovered and showed to this Society many years since, which of late Mr. Newton has done me the favor to print and publish as his own inventions” (Hall 1951, 224). There's no evidence that Hooke could have ever worked out the mathematics of gravity or its application to celestial mechanics. Yet Newton was forced to acknowledge that Hooke had provided him with some insights (Deming 2012, 238).

## Capillary Action

In 1647, a German engineer, Otto von Guericke (1602–2686) invented the air pump. Ten years later, at Magdeburg, Germany, Guericke demonstrated the existence of a vacuum and the power of air pressure by evacuating two halves of a hollow copper sphere. After air had been removed from the sphere, a team of horses was not strong enough to separate the two halves (Deming 2016, 88).

At Oxford, Robert Boyle (1627–1691) learned of Guericke's experiments with the air pump and was fascinated by the possibility of conducting a wide range of scientific experiments. Boyle commissioned Ralph Greatrex, an accomplished London fabricator of precision instruments to construct an air pump for him. But Greatrex was evidently unable to fabricate the machine to Boyle's satisfaction (Deming 2016, 89). Boyle was introduced to Robert Hooke by Thomas Willis (1621–1675) (Westfall 2008, 482). Hooke succeeded in constructing an air pump to Boyle's specifications and became Boyle's collaborator in a series of groundbreaking experiments.

Boyle and Hooke's experiments with the air pump were published in 1660 under the title *New Experiments Physico‐Mechanical* (Boyle 1660). Boyle and Hooke discovered *Boyle's law*, the observation that the pressure exerted by a gas is inversely proportional to the volume it occupies. But their work was far more significant than a single technical contribution to physical chemistry. The methodology of Boyle and Hooke virtually defined the practice of what they would have termed experimental philosophy. Today, we refer to it as *science*. Among the methodological insights found in *New Experiments Physico‐Mechanical* are a recognition of the provisional nature of scientific knowledge, the criterion of repeatability, and an anticipation of Chamberlin's method of multiple working hypotheses (Deming 2016, 91–93).

*New Experiments Physico‐Mechanical* (Boyle 1660, 267–272) contains one of the first descriptions of the phenomenon of capillary action. Boyle recounts that he was told by “an eminent mathematician” that if “one end of a slender and perforated pipe of glass be dipped in water, the liquor will ascend to some height in the pipe” (Boyle 1660, 267–268). Fascinated by this phenomenon, Boyle, with the probable assistance of Hooke, repeated the experiment and found “if the pipes were made slender enough, the water might rise to a very much greater height” (Boyle 1660, 268). Among the variations considered by Boyle and Hooke were inclining the capillary tubes, fashioning some of them with bends resembling siphons, and substituting red wine for water. The results of these experiments excited the “wonder of some famous mathematicians” (Boyle 1660, 268). But a satisfactory explanation was lacking. Boyle concluded that “the cause of this ascension of the water, appeared to all that were present so difficult, that I must not stay to enumerate the various conjectures that were made” (Boyle 1660, 269).

Hooke returned to the subject of capillary action in his first publication, *An Attempt for the Explication of the Phaenomena* (1661). Hooke conducted a number of experiments with capillary tubes of various diameters and attempted to formulate a hypothesis to explain the phenomenon. He postulated that matter exhibited a property of “congruity” or “incongruity” that manifested as an attraction or repulsion between different species of materials. As capillary action is due to molecular forces, this is, in a liberal sense, the correct explanation (Deming 2002, 145). Hooke maintained that “there is a much greater inconformity or incongruity of air to glass … than there is of water to the same” (Hooke 1661, 7). But after recognizing the existence of molecular forces, Hooke concluded incorrectly that the rise of water in capillary tubes was caused by a lower air pressure in the tubes. “There is a greater pressure of the air upon the water in the vessel or greater pipe, than there is upon that in the lesser pipe” (Hooke 1661, 25). This conclusion was repeated in *Micrographia* (Hooke 1665, 11).

Hooke's theory was generally accepted for a few decades. Cambridge mathematician Roger Cotes (1682–1716) noted that “Dr. Hooke's hypothesis has indeed the fairest show of probability, and accordingly it has been received with great applause” (1738, 116). But Cotes himself falsified Hooke's theory by means of a simple experiment: he observed that capillary action occurred even in a vacuum. Thus the cause could not be differential air pressure. “Even under a receiver exhausted of air by the air‐pump, there is as far as we can perceive a like and equal ascent of liquors in capillary tubes” (Cotes 1738, 118–119). Cotes concluded simply that the true cause of capillary action was the existence of an attractive force of unknown origin between water and glass (Cotes 1738, 122). In 1805, Thomas Young (1773–1829) and Pierre Simon Laplace (1749–1827) described capillary action mathematically with the derivation of the Young‐Laplace equation (Brewster 1832, 812).

Where Hooke was insightful was in recognizing that the phenomenon of capillary action had implications for biological and geological processes. He speculated that capillary action might well be involved in “the ascending of the sap in trees and plants, through their small, and some of them imperceptible pores,” as well as the passage of water from the soil to plant roots (Hooke 1661, 26).

In the latter half of the seventeenth century, the nature of the hydrologic cycle was still in dispute. The ancient idea that springs and rivers were recharged by some hidden percolation from the oceans back to the land remained a viable hypothesis (Deming 2014, 2018). The concept persisted until Edme Mariotte published calculations in 1686 showing that rainfall in the Seine Basin was more than sufficient to account for flow in the river (Deming 2018).

There were two problems with the theory that streams were recharged by sea water passing through the Earth. First, flow from the sea to the land was uphill. If it occurred, there must be some propelling force or mechanism. Second, sea water was salty, but terrestrial streams were fresh. The existence of capillary action suggested a possible way for water to flow uphill. Hooke raised the question of “whether the rising and ebullition of the water out of springs and fountains may not be explicated by the rising of water in a smaller pipe: for the sea water being as it were strained through the pores or crevices of the Earth, is as it were included in little pipes” (Hooke 1661, 31–32). To explain the loss of salt, Hooke suggested that the salt might be removed by a chemical reaction. “Some parts of the Earth through which it is to pass, may contain a salt, that mixing and uniting with the sea salt, may precipitate it” (Hooke 1661, 34).

But Hooke held no theory dogmatically. After examining the possible relevance of capillary action to the hydrologic cycle, he criticized the same theory. Hooke raised the question: if terrestrial springs were recharged by sea water, then why is it that “springs do not run faster and slower, according to … the ebbing and flowing of the sea?” (Hooke 1661, 36). An experimental philosopher was never dogmatic or close‐minded. Hooke explained, “I neither conclude from one single experiment, nor are the experiments I make use of, all made upon one subject. Nor wrest I any experiment to make it *quadrare* with any preconceived notion. But on the contrary, I endeavor to be conversant in all kind of experiments” (Hooke 1661, 41).

## Hydrologic Cycle

As noted before, in the late seventeenth century the question of whether precipitation was sufficient to fully account for terrestrial stream flow was still in dispute. In *Lectures De Potentia Restitutiva* (1678), Hooke gave his thoughts on the subject. First, he noted existence of the hydrologic cycle. Due to the influence of heat and wind, water could be “reduced into the form of air,” rise into the upper atmosphere, and there “condense and revert into water” (Hooke 1678, 36). Hooke would have had no way of knowing that air was composed of a number of chemically discrete gases, all of them invisible, odorless, and colorless. Since at least the time of Aristotle, evaporation had been considered to be the transformation of water into air.

Hooke admitted “it remains a difficulty that all rivers and springs should have their original from the water that falls or condenses out of the air,” but proceeded to cite a number of arguments as to why he believed this to be the case (Hooke 1678, 36).

First, Hooke noted that “great inundations or overflowing of rivers manifestly proceed either from the rain that immediately falls, or from the melting of snow or ice that has formerly fallen on the more eminent parts of mountains” (Hooke 1678, 37). Second, Hooke claimed that quantitative calculations had been done to demonstrate that “there falls water enough from the sky in actual rain, snow, or hail upon the surface of England to supply all the water that runs back into the sea by rivers” (Hooke 1678, 37). If true, these calculations were done at least 8 years before the publication of Edme Mariotte's work in *Traité du mouvement des eaux et des autres corps fluide* (Mariotte 1686). Unfortunately, Hooke provided no specifics.

Pierre Perrault (1611–1680) published calculations that were similar to those of Mariotte in *De l'origine des fontaines* (*The Origin of Springs*) (Perrault 1674). Thus Perrault predated both Hooke and Mariotte (Deming 2014). However, Mariotte continues to be credited as the first person to quantitatively demonstrate the sufficiency of precipitation to account for all stream flow because his calculations were more conclusive than those done by Perrault. Yet if Hooke can be believed, Mariotte was anticipated in England by several years.

Third, Hooke noted that the only known way of producing fresh water from salt water was by distillation. Therefore “there is little probability that the springs at the top of a high hill should proceed from the seawater strained through the earth” (Hooke 1678, 37). Fourth, Hooke noted the certain and universal existence of evaporation and precipitation. The fifth reason was an observation that water “is condensed out of the air by the trees at the tops of the hills” (Hooke 1678, 38). The sixth argument was the existence of “many springs” near the bottoms of “high hills” containing “very fresh and clear water,” and this was attributed to “the air being dashed and broken” against the hills (Hooke 1678, 38).

## Contributions to Geology

In late seventeenth century Europe, it was commonly supposed that the Earth was no more than a few thousand years old. The primary source for its chronology was the *Bible*. In 1650 Anglican Bishop James Ussher (1581–1656) published *Annales Veteris Testamenti, a Prima Mundi Origine Deducti* (*The Annals of the World Deduced from the Origin of Time*) wherein he asserted that “the beginning of time … fell upon the entrance of the night preceding the twenty‐third day of October” in the year 4004 BC (Ussher 1650, 1658, 1). Nor was Ussher's view unique. No less a person than Isaac Newton penned a scholarly young‐Earth history, *The Chronology of Ancient Kingdoms Amended* (Newton 1728).

Corollaries to the young‐Earth chronology included the idea that the Earth was static. According to the book of *Genesis*, God had separated the sea and the land on the third day of creation. There was no concept that the sea and land had changed place. Biological extinction was also a foreign idea. “Once God had established His perfect order within the natural world, it was inconceivable that He would allow that natural order to be destroyed by a species becoming extinct” (Rowland 2009, 227). As late as 1799, Thomas Jefferson (1743–1826) argued that admitting to the reality of extinction would unravel the providential natural order established by God. “If one link in nature's chain might be lost, another and another might be lost, till this whole system of things should evanish by piece‐meal” (Jefferson 1799, 255–256).

In 1669 a Danish anatomist published a curious pamphlet with the odd title *De solido intra solidum naturaliter contento dissertationis prodromus* (*Prodromus to a Dissertation on Solids Naturally Enclosed in Solids*) (Steno 1669). In *De Solido* Nicolaus Steno (1638–1686) proposed that the history of the Earth was to be found in the Earth itself, and postulated three rules of geologic interpretation (Cutler 2003). For this reason, Steno is given substantial credit as a founder of the modern science of geology. Yet many of Steno's conclusions were mirrored by Robert Hooke. One of Hooke's biographers has argued that “in several ways his [Hooke's] ideas were more advanced than those of Steno” (Oldroyd 1972, 109).

Most of Robert Hooke's ideas were presented in a series of lectures to the Royal Society in 1667 and 1668 (Oldroyd 1972, 110). If true, that would predate the publication of Steno's *Prodromus* in 1669. But the lectures were only published posthumously in 1705, and the editor, Richard Waller, noted that Hooke's lectures were “made and read by him at several distant times” (Waller 1705b). There is some evidence that Hooke may have made the claim that the secretary of the Royal Society, Henry Oldenburg (1619–1677), transmitted his ideas to Steno in Italy (Inwood 2003, 351). As an English translation of *De Solido* appeared in 1671, it seems certain that Steno's pamphlet of 1669 was known and discussed in London soon after its publication (Steno 1671).

**Figure 1:**
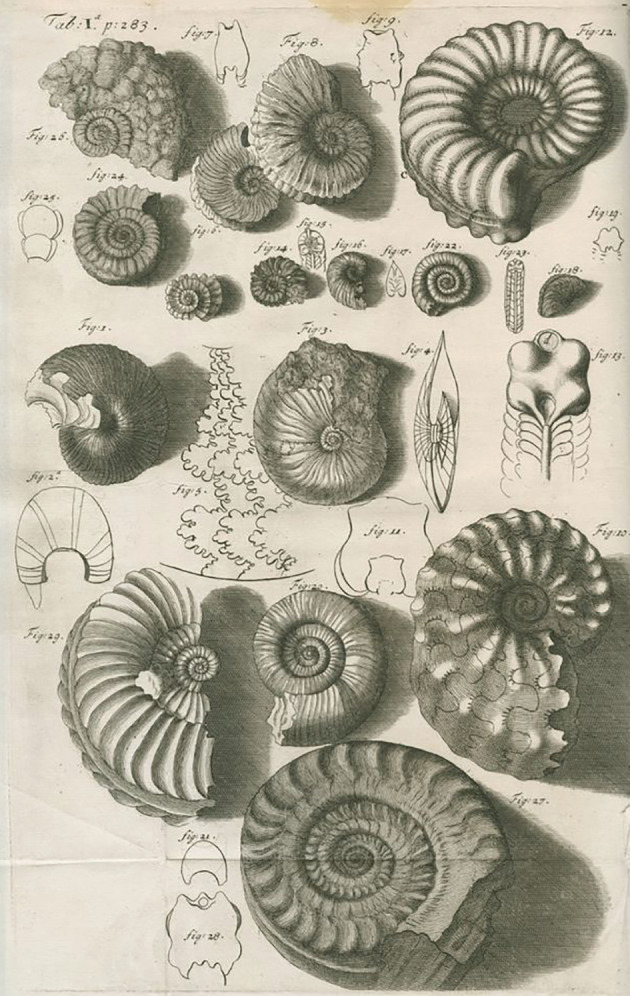
Fossil shells drawn by Hooke (1705, 283).

**Figure 2:**
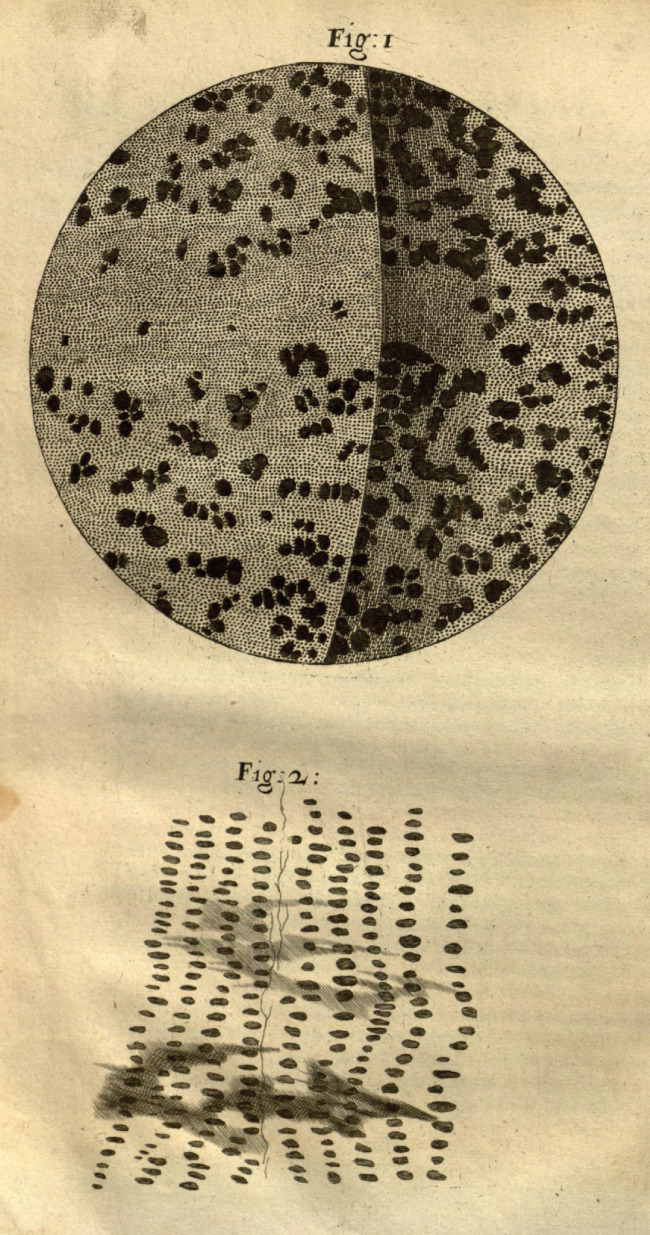
Pores in “coal or wood charred” (top), and petrified wood (bottom) drawn by Robert Hooke. Hooke's microscopic examination of wood and petrified wood helped convince him that fossils were the petrified remains of living creatures (Hooke 1665, 107).

Hooke's geological ideas were important and revolutionary (Geike 1905). First, Hooke argued that fossils, or “figured stones,” were in fact the remains of living organisms turned to stone through a process of petrification (Figure 1). It is certain that Hooke's interest in petrification predated the publication of Steno's *De Solido* in 1669, because *Micrographia* (Hooke 1665, 107–112) contains a discussion of both petrified wood and shells. It is not difficult to understand how Hooke came to believe in the reality of petrification when we read his description of how a piece of petrified wood under magnification exhibited striking similarities to wood (Figure 2). “All the smaller microscopical pores of it appear perfectly like the microscopical pores of several kinds of wood … retaining both the both, position and magnitude of such pores” (Hooke 1665, 108).

Throughout the Middle Ages and the Renaissance the origin of fossils was debated. Some writers argued that fossils arose “as the works of an occult power or influence at work in nature” (Adams 1954, 250). The existence of the magnet, by which objects could be moved at distance through no physical mechanism, was undeniable proof that nature contained hidden forces. Hooke acknowledged these theories by noting that some people believed fossils were formed “by some plastic virtue inherent in those parts of the Earth … or else by some celestial influence or aspect of the planets operating at a distance upon the yielding matter of the parts of the Earth” (Hooke 1705, 288). As “figured stones” were found on the highest mountaintops, it was difficult for people in the seventeenth century to accept that these were the petrified remains of sea creatures. The Catholic priest and metallurgist, Alvaro Alonso Barba (1569–1662), who worked mines in South America, argued that it was “madness to imagine that ever the sea has prevailed” in mountainous regions (Barba 1674, 68).

Others argued that petrification was a reality. As early as the first century AD, Pliny the Elder (AD 23–79) noted that the existence of stalagmites and stalactites in caves was evidence that water could petrify (Pliny the Elder 1856, 482). And in 1580, Bernard Palissy (c. 1510–1589) asserted that “not only wood may be turned to stone, but also the body of man and beast” (Palissy 1957, 151). But the origin of fossils remained a subject of active debate through the end of the seventeenth century. In 1671 Martin Lister (1639–1712) published a letter in the *Transactions of the Royal Society* where he argued that “there is no such matter as petrifying of shells” (Lister 1671, 2282).

Hooke asserted unambiguously that he believed “the greatest part” of “figured bodies” to be “animal or vegetable substances converted into stone by having their pores filled up with some petrifying liquid substance” (Hooke 1705, 290). Furthermore, he described fossils as the key to uncovering the history of the Earth. “These [fossils] are the greatest and most lasting monuments of antiquity, which, in all probability, will far antedate all the most ancient monuments of the world, even the very pyramids, obelisks, mummies, hieroglyphics, and coins, and will afford more information in natural history, than those other put together will in civil” (Hooke 1705, 335). Hooke thus anticipated Georges Cuvier (1769–1832), who first established that the key to unraveling the history of the Earth lay not so much in rocks as in fossils (Rudwick 1997).

Nevertheless, Hooke's reference to pyramids and other human artifacts indicates he was not yet thinking in terms of millions or billions of years. He admitted that the eventual establishment of a chronology from fossils would be a difficult work. “It must be granted, that it is very difficult to read them, and to raise a chronology out of them” (Hooke 1705, 411). Hooke concluded that in his time there was “no other means of being informed of the true history of it [the Earth], but what is to be found recorded in the sacred writings of Moses” (Hooke 1705, 412).

Hooke's colleague in the Royal Society, Martin Lister, did not believe in petrification because many of the fossils he examined were “quite different … not only from one another … [but] from anything in nature besides, that either the land, salt, or fresh water does yield us” (Lister 1671, 2283). Hooke countered with the argument that perhaps some or many of the fossilized species were extinct. “There have been many other species of creatures in former ages” (Hooke 1705, 291). And he explicitly postulated the corollary of new creation. “It is not unlikely also but that there may be diverse new kinds now, which have not been from the beginning” (Hooke 1705, 291). If we ascribe naturalism to Hooke, biological evolution is implied. Hooke explicitly postulated that in addition to the possibility “that there may have been diverse species of things wholly destroyed and annihilated,” it was also likely that there were “diverse others changed and varied” (Hooke 1705, 327). However, he did not admit change to the point of speciation but rather spoke of “new varieties generated of the same species” (Hooke 1705, 327).

Hooke countered the idea of a static Earth with the argument that the sea and land had changed places in the distant past. It was not a new idea. In *Meteorologica*, Aristotle (384–322 BC) had asserted that “the world is eternal,” and that “if the sea is always advancing in one place and receding in another it is clear that the same parts of the whole Earth are not always either sea or land, but that all this changes in course of time” (Aristotle 1923, 353a).

Hooke proposed “that a great part of the surface of the Earth has been since the Creation transformed and made of another nature; namely, many parts which have been sea are now land, and diverse other parts are now sea which were once a firm land” (Hooke 1705, 290). The naturalistic and uniformitarian mechanism Hooke invoked to explain the transformation of the Earth's surface was earthquakes. Earthquakes could raise the seabed above land (Hooke 1705, 299–300), create mountains from plains (Hooke 1705, 302), and generally “transpose, covert, subvert and jumble the parts of the Earth together” (Hooke 1705, 309). The modification of the globe by seismic action was alleged to be a universal and active principle (Hooke 1705, 311). Acting in opposition to the uplift of land and sea by earthquakes was the continual eroding action of gravity in concert with wind and water (Hooke 1705, 304).

While Hooke argued that the sea and land had changed place in the past, he also acknowledged that no significant changes had taken place during the few thousand years of recorded human history. “All places, countries, seas, rivers, islands, etc. have all continued the same for so long time as we can reach backwards with any history” (Hooke 1705, 416). Thus Hooke's invocation of a uniformitarian mechanism to explain geologic changes necessarily and implicitly falsified the concept of a young Earth.

As late as the first decades of the nineteenth century, the Biblical Deluge was invoked by leading geologists as a major agent of geologic change. In *Reliquiae Diluvianae*, William Buckland (1784–1856) attributed the presence of fossil bones in English caves to the action of a universal deluge (Buckland 1823). Hooke was not yet ready to entirely discard the Biblical Deluge as false, but he undercut it by arguing that the Biblical Flood narrative was inadequate to explain the presence of fossilized seashells on mountaintops. These shells, he explained, “could not be from the Flood of Noah, since the duration of that which was but about two hundred natural days” (Hooke 1705, 3410).

## Legacy

The Royal Society was founded in 1660 and received their charter in 1662 (Hall 2008). Its activities came to virtual define science as we understand and practice it today. And one of the key factors that maintained the Society's initiative was the appointment of Robert Hooke as “curator of experiments” in 1662. Science was experimental philosophy, and it was Hooke's duty to “furnish them every day when they met, with three or four considerable experiments” (Waller 1705a, ix). Richard Westfall (1924–1996) argued that “it is hard to imagine that the Royal Society would have survived the apathy that succeeded its initial burst of enthusiasm without the stimulus of Hooke's experiments, demonstrations, and discourses” (Westfall 2008, 483). Thus Robert Hooke was a key figure in the founding of modern science, in more ways than one.

Hooke was a transitional figure. He undermined, but was not yet willing to abandon the Biblical chronology. His theories were mechanistic, he never invoked metaphysical explanations, and he was completely devoted to empiricism. Yet Hooke's writings and work are replete with the sort of unbridled speculations that led natural philosophy to a sterile dead end. Hooke was infamous for never being able to complete his scientific work in a rigorous and thorough manner. He “hurried from one inquiry to another with brilliant but inconclusive results” (Clerke 1891, 284).

Typical of Hooke's methodology was his attempt to detect stellar parallax. In the summer and fall of 1669, Hooke made observations of the star *Gamma Draconis* using a zenith sector, a telescope designed to point directly at the zenith point, 90° from the horizontal. Hooke measured the position of *Gamma Draconis* on two nights in July, and followed up with two nights of observation the following October. From these scant observations, Hooke triumphantly announced positive results and declared that he had detected parallax amounting to “27 or 30 seconds” of arc (Hooke 1674, 26).

Had Hooke been more perspicacious, he would have realized that the “parallax” he had observed was in the wrong direction. From a lengthy series of meticulous and precise observations, James Bradley (1693–1762) later discovered that the apparent movement of *Gamma Draconis* was caused by an entirely hitherto unsuspected phenomenon: the aberration of light. Bradley's patient and thorough methods enabled him to (1) confirm the Copernican hypothesis, (2) accurately estimate the speed of light, and (3) expand the physical scale of the visible universe. For this work, Freeman Dyson (b. 1923) called James Bradley “the inventor of modern science” because he “was the first to understand that accurate measurement requires meticulous monitoring and control of possible sources of error” (Dyson 1999, 27). Standing with one foot in the Middle Ages, Hooke apparently never realized that the experimental philosophy he embraced required a commitment to new standards of precision and reliability.

In the late 1690s, Hooke's health began to decay. He suffered from scurvy, went blind, and became bedridden (Waller 1705a, xxvi). Decrepit as his body was, Richard Waller assures us that Hooke retained his “active, restless, indefatigable genius even almost to the last” (Waller 1705a, xxvii). Hooke died on the third day of March, 1703, in the most abject circumstances. His body was covered with lice and “ragged clothes were twisted about his emaciated body” (Inwood 2003, 3). Hooke never bothered to compose a will, and his possessions were those of a poor man. Yet a search of his house found “a large iron chest of money … to the value of many thousands in gold and silver” (Waller 1705a, xiii). This fortune, equivalent to about a million English pounds in modern currency (Inwood 2003, 4), had apparently been accumulated by Hooke when he had been employed as an architect during the rebuilding that followed the great London fire of 1666. Miserly, Hooke perished in miserable circumstances.

Hooke's epitaph was written by Richard Waller. “All his errors and blemishes were more than made amends for, by the greatness and extent of his natural and acquired parts, and more than common, if not wonderful sagacity, in diving into the most hidden secrets of nature” (Waller 1705a, xxviii).

## References

[gwat12849-bib-0001] Adams, F.D. 1954 The Birth and Development of the Geological Sciences. New York: Dover.

[gwat12849-bib-0002] Aristotle . 1923 Meteorologica, translated by E. W. Webster. London, UK: Oxford University Press.

[gwat12849-bib-0003] Aubrey, J. 1898 Brief Lives, Vol. 1 Oxford, UK: Clarendon Press.

[gwat12849-bib-0004] Bacon, F. 1620 Francisci de Verulamio, Summi Angliæ Cancellarii, Instauratio Magna (Novum Organum). London, UK: Apud Bonham Norton and Ioannem Billium Typographum Regium.

[gwat12849-bib-0005] Barba, A.A. 1674 The Art of Metals, translated by the Earl of Sandwich. London, UK: S. Mearne.

[gwat12849-bib-0006] Barker, G.R.R. 1895 Memoir of Richard Busby. London, UK: Lawrence and Bullen.

[gwat12849-bib-0007] Birch, T. 1744 The life of the Honourable Robert Boyle In The Works of the Honourable Robert Boyle, Vol. 1, 1–139. London, UK: A. Millar.

[gwat12849-bib-0008] Boyle, R. 1660 New Experiments Physico‐Mechanical, Touching the Spring of the Air. Oxford, UK: H. Hall.

[gwat12849-bib-0009] Brewster, D. 1832 Hydrodynamics In The Edinburgh Encyclopedia, Vol. 10, 751–909. Philadelphia, Pennsylvania: Joseph and Edward Parker.

[gwat12849-bib-0010] Buckland, W. 1823 Reliquiae Diluvianae. London, UK: John Murray.

[gwat12849-bib-0011] Clerke, A.M. 1891 Hooke, Robert In Dictionary of National Biography, ed. Lee Sidney, 283–287. New York: Macmillan.

[gwat12849-bib-0012] Cotes, R. 1738 Hydrostatical and Pneumatical Lectures. London, UK: Robert Smith.

[gwat12849-bib-0013] Cutler, A. 2003 The Seashell on the Mountaintop. New York: Dutton.

[gwat12849-bib-0014] Deming, D. 2018 Edme Mariotte and the beginning of quantitative hydrogeology. Groundwater 56, no. 2: 350–355. 10.1111/gwat.12609 29120496

[gwat12849-bib-0015] Deming, D. 2016 Science and Technology in World History, *Vol. 4*, *The Origin of Chemistry, the Principle of Progress, the Enlightenment and the Industrial Revolution*. Jefferson, North Carolina: McFarland.

[gwat12849-bib-0016] Deming, D. 2014 Pierre Perrault, the hydrologic cycle and the scientific revolution. Groundwater 52, no. 1: 156–162. 10.1111/gwat.12138 24224605

[gwat12849-bib-0017] Deming, D. 2012 Science and Technology in World History*, Vol. 3*, *The Black Death, the Renaissance, the Reformation, and the Scientific Revolution*. Jefferson, North Carolina: McFarland.

[gwat12849-bib-0018] Deming, D. 2002 Introduction to Hydrogeology. New York: McGraw‐Hill.

[gwat12849-bib-0019] Dyson, F.J. 1999 The inventor of modern science. Nature 400, no. 6739: 27.

[gwat12849-bib-0020] Geike, A. 1905 The Founders of Geology. London, UK: Macmillan.

[gwat12849-bib-0021] Gould, R.T. 1935 John Harrison and his timekeepers. Mariner's Mirror 21, no. 2: 115–139.

[gwat12849-bib-0022] Hall, M.B. 2008 Royal Society of London In Encyclopedia of the Scientific Revolution, ed. Applebaum Wilbur, 582–585. New York: Routledge.

[gwat12849-bib-0023] Hall, A.R. 1951 Two unpublished lectures of Robert Hooke. Isis 42, no. 3: 219–230. 14880332 10.1086/349309

[gwat12849-bib-0024] Halley, E. 1700 An account of Dr. Robert Hooke's invention of the marine barometer. Philosophical Transactions of the Royal Society of London 22, no. 269: 791–794.

[gwat12849-bib-0025] Hooke, R. 1705 In A Discourse of Earthquakes, ed. Waller Richard London, UK: Sam. Smith and Benjamin Walford.

[gwat12849-bib-0026] Hooke, R. 1678 Lectures De Potentia Restitutiva. London, UK: Printed for John Martyn.

[gwat12849-bib-0027] Hooke, R. 1676 A Description of Helioscopes. London, UK: Printed by T. R. for John Martyn.

[gwat12849-bib-0028] Hooke, R. 1674 An Attempt to Prove the Motion of the Earth from Observations. London, UK: Printed by T. R. for John Martyn.

[gwat12849-bib-0029] Hooke, R. 1665 Micrographia. London, UK: Printed by Jo. Martyn and Ja. Allestry.

[gwat12849-bib-0030] Hooke, R. 1661 An Attempt for the Explication of the Phaenomena. London, UK: J. H. for Sam Thomson.

[gwat12849-bib-0031] Inwood . 2003 The Forgotten Genius. San Francisco, California: MacAdam/Cage.

[gwat12849-bib-0032] Jefferson, T. 1799 A memoir on the discovery of certain bones of a quadruped of the clawed kind in the western parts of Virginia. Transactions of the American Philosophical Society 4: 246–260.

[gwat12849-bib-0033] Lister, M. 1671 A letter of Mr. Martin Lister. Philosophical Transactions of the Royal Society of London 6, no. 76: 2281–2284.

[gwat12849-bib-0034] Mariotte, E. 1686 Traité du mouvement des eaux et des autres corps fluide. Paris: Chez E. Michallet.

[gwat12849-bib-0035] McConnell, A. 2005 Origins of the marine barometer. Annals of Science 62, no. 1: 83–101.

[gwat12849-bib-0036] Newton, I. 1960 The Correspondence of Isaac Newton, Vol. 2 London, UK: Cambridge University Press.

[gwat12849-bib-0037] Newton, I. 1728 The Chronology of Ancient Kingdoms Amended. London, UK: J. Tonson.

[gwat12849-bib-0038] Oldroyd, D.R. 1972 Robert Hooke's methodology of science as exemplified in his “discourse of earthquakes”. The British Journal for the History of Science 6, no. 2: 109–130.

[gwat12849-bib-0039] Overton, J.H. 1886 Busby, Richard In Dictionary of National Biography, Vol. 8, ed. Stephen Leslie, 29–31. London, UK: Smith, Elder & Co.

[gwat12849-bib-0040] Palissy, B. 1957 The Admirable Discourses of Bernard Palissy, translated by Aurèle La Rocque. Urbana, Illinois: University of Illinois Press.

[gwat12849-bib-0041] Perrault, P. 1674 De l'origine des fontaines. Paris: Pierre le Petit.

[gwat12849-bib-0042] Pliny the Elder . 1856 The Natural History of Pliny, Vol. 5, translated by John Bostock and H. T. Riley. London, UK: Henry G. Bohn.

[gwat12849-bib-0043] Rowland, S.M. 2009 Thomas Jefferson, extinction, and the evolving view of earth history in the late eighteenth and early nineteenth centuries In Geological Society of America Memoir 203*, The Revolution in Geology from the Renaissance to the Enlightenment*, ed. Rosenberg G.D., 224–246. Boulder, Colorado: Geological Society of America.

[gwat12849-bib-0044] Rudwick, M.J.S. 1997 Georges Cuvier, Fossil Bones, and Geological Catastrophes. Chicago, Illinois: University of Chicago Press.

[gwat12849-bib-0045] Stone, L. 1977 The Family, Sex and Marriage in England 1500–1800. New York: Harper & Row.

[gwat12849-bib-0046] Steno, N. 1671 The Prodromus to a Dissertation Concerning Solids Naturally Contained Within Solids *,* translated by H.O. London, UK: F. Winter.

[gwat12849-bib-0047] Steno, N. 1669 Nicolai Stenonis De Solido Intra Solidum Naturaliter Contento Dissertationis Prodromus. Florence, Italy: Ex typographia sub signo stell.

[gwat12849-bib-0048] Ussher, J. 1658 The Annals of the World Deduced From the Origin of Time. London, UK: Printed by E. Tyler for J. Crook.

[gwat12849-bib-0049] Ussher, J. 1650 Annales Veteris Testamenti, a Prima Mundi Origine Deducti. London, UK: J. Flesher, J. Crook, & J. Baker.

[gwat12849-bib-0050] Waller, R. 1705a The Life of Dr. Robert Hooke In The Posthumous Works of Robert Hooke, i–xxviii. London, UK: Sam. Smith and Benj. Walford.

[gwat12849-bib-0051] Waller, R. 1705b The Publisher to the Reader In The Posthumous Works of Robert Hooke. London, UK: Sam. Smith and Benj. Walford.

[gwat12849-bib-0052] Westfall, R.S. 2008 Hooke, Robert In Complete Dictionary of Scientific Biography, Vol. 6, ed. Gillispie Charles Coulston, 481–488. Detroit, Michigan: Charles Scribner's Sons.

